# Collective chemotaxis and segregation of active bacterial colonies

**DOI:** 10.1038/srep21269

**Published:** 2016-02-18

**Authors:** M. Ben Amar

**Affiliations:** 1 Laboratoire de Physique Statistique, Ecole Normale Supérieure, UPMC Univ Paris 06, Université Paris Diderot, CNRS, 24 rue Lhomond, 75005 Paris, France; 2 Institut Universitaire de Cancérologie, Faculté de médecine, Université Pierre et Marie Curie-Paris 6, 91 Bd de l’Hôpital, 75013 Paris, France

## Abstract

Still recently, bacterial fluid suspensions have motivated a lot of works, both experimental and theoretical, with the objective to understand their collective dynamics from universal and simple rules. Since some species are active, most of these works concern the strong interactions that these bacteria exert on a forced flow leading to instabilities, chaos and turbulence. Here, we investigate the self-organization of expanding bacterial colonies under chemotaxis, proliferation and eventually active-reaction. We propose a simple model to understand and quantify the physical properties of these living organisms which either give cohesion or on the contrary dispersion to the colony. Taking into account the diffusion and capture of morphogens complicates the model since it induces a bacterial density gradient coupled to bacterial density fluctuations and dynamics. Nevertheless under some specific conditions, it is possible to investigate the pattern formation as a usual viscous fingering instability. This explains the similarity and differences of patterns according to the physical bacterial suspension properties and explain the factors which favor compactness or branching.

During the last decades, experimental set-ups of dilute bacterial colony have demonstrated a diversity of complex and ramified patterns, presenting similarities with viscous fingering fronts. The literature on these patterns is very rich, going from pioneering works by Ben Jacob and collaborators^1^,^2^ to more recent publications^3^,^4^,^5^ and the present biology allows to multiply the possibilities with mutants. Adopting a macroscopic viewpoint, valid on scales larger than the bacterium size, we aim to explain this diversity observed in experiments, also shown in simulations. The scope is to understand the physical and biological features at the origin of extremely ramified patterns when growth, chemotaxis and hydrodynamics are coupled.

Recently a lot of experimental works^6^,^7^ concern active bacteria, having their own dynamics which modifies the averaged flow^8^. It appears that most of the physical laws concerning dilute solutions may be or must be revisited in the case of active particles. Depending on their densities, at the macro-scales, they induce bizarre rheology^9^,^10^,^11^,^12^, hydrodynamic instabilities^13^, collective motion and patterns, and also complex chaotic or turbulent flows^14^. For example, a thin film containing “pusher bacteria” exhibits rolls above a critical thickness^9^ demonstrating that a vertical chemotactic flux can play the role of the temperature gradient in the classical Bénard experiment as explained in^8^,^15^. These active properties exacerbate the coupling with chemotaxis, known to produce instabilities on colony fronts^16^,^17^.

Unlike most of the recent literature on active bacteria mostly devoted to collective behavior in forced flows, a minimal model is proposed for the colony expansion in radial geometry eventually driven by chemotaxis. This continuous model couples bacterial density and velocity via the mass flux equation including eventually chemo-attractants, nutriments or by-products, our scope being to explain the observed patterns in analogy with viscous fingering. In the radial geometry, commonly chosen in experiments and simulations, this similarity appears clearly, the evolving pattern being circular at short times then evolving with more or less compact fingering patterns.

Early treatments on chemotactic bacterial patterns were based on reaction-diffusion, and, hydrodynamics was discarded or treated as a perturbation of the density variation^18^,^19^,^20^. Such hypothesis cannot be maintained in growth as shown in^21^. The opposite simplifying limit consists in assuming that the advection/proliferation drives the formation of growing iso-density domains as suggested by Greenspan^22^ in tumorigenesis and many others since then^23^,^24^. However, as shown explicitly here, iso-density colony with sharp density gap between domains, cannot exist under chemotaxis or auto-chemotaxis. This property is not limited to bacterial colonies but also concerns any self-assembly of living matter. Indeed, chemotaxis is a a crucial mechanism for collective motion such as cell migration^25^,^26^,^27^, cancer metastasis^28^,^29^, wound-healing^30^,^31^, and any inflammatory process^32^. The model presented here can be easily adapted to any 2-dimensional colony migration and the diffuse zone at the border of domains also applies to moving epithelia as for bacterial colonies. As a consequence, any diffuse front of living colonies strongly suggests the presence of morphogen gradients. For a moving epithelium, the cells at the border can be more or less flattened like observed in^33^, sometimes the front itself becomes noisy as in^34^; for bacteria in solution, the density weakens smoothly at the front of separation, creating a density boundary layer whose stability is not completely guaranteed at long times and prevents any analogy with viscous fingering. Nevertheless, when the boundary layer remains stable and confined, eventually for particular cellular proliferation rates, the model proposed here explain the observed pattern dynamics under the simultaneous coupling of growth, chemotaxis, rheology, and cellular activity^35^. Analytical insights are restricted to short time scales but allow a better understanding on the interactions at the origin of the pattern diversity demonstrated in^1^,^2^, as example.

## The Model

### The main equations

Bacteria are very sensitive to morphogens which bias their random walk and orient their averaged motion. Some are attractant, others are repellant, they may be also nutrients. In addition, they can be produced by the bacteria themselves (auto-chemotaxis). The cells migrate according to their local gradient (along the radius in circular geometry). Cellular velocities being very small, the chemotactic flux 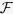 is given by 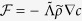, with 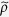 the cellular concentration and 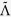 being positive or negative according to the morphogenetic signaling. Morphogen concentrations and their gradients are difficult to control in experiments since bacteria have also the possibility to produce them. We then consider that they are controlled, far away from the bacterial domain (called 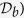, at the border of the experimental set-up to a value 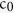. Taking 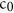 as concentration unit, 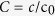 satisfies a simple diffusion-consumption equation in 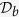 and a pure diffusion equation in 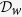, the domain which is not reached by the bacterial front. Choosing, as time unit, 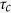 the typical time for the morphogen capture by a colony of density 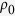 (also taken as density unit hereafter), the length unit is then given by 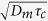 with 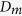 the diffusion coefficient for dilute colony, assumed identical in 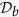 and 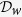. So the concentration field satisfies the dimensionless equation:


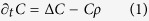


with 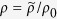. Defining 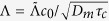, the mass flux equation for the bacterial population is given by


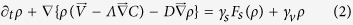


The left-hand side of Eq(2) is rather classical, takes into account the advection term 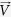, the chemotactic forcing 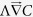 and the density variation while the right-hand side comes from the bacteria proliferation. The difference between the standard mass-flux equation of hydrodynamics for compressible fluids and Eq.(2) only comes from the chemotactic term (left-hand-side) and the proliferation rate (right-hand-side).

**Figure 1:**
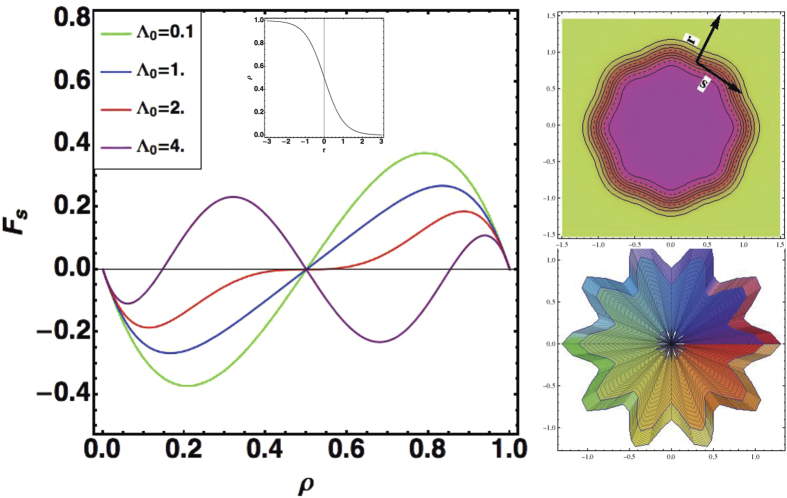
Proliferation rate *F*_*s*_ corresponding to Eq. (2) for different values of the chemotactic parameter Λ_0_ defined below Eq.(6). Density variation at a circular border in the inset. On top and right, the colony density variation profile (pink corresponds to a bacterial density value equal to 1, green to 0). The top figure corresponds to an instability of the density boundary layer. Below, a typical pattern obtained when the bulk is unstable.

Let us discuss the choice made for the proliferation rate. With an experimental set-up saturated with nutrients, the colony will tend to maintain its density to an average value (homeostatic state) and simultaneously will expand which requires cellular proliferation. So in 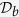, the cellular density will be close to a characteristic value 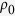, hereafter taken as density unit, but at the border, a more or less abrupt jump exists that will trigger an intense and localized proliferation. This is mathematically obtained by choosing 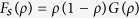 giving a surface proliferation rate which vanishes in both domains: 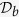 and 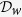 so for 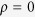 and 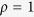 [ref] 18. The volume growth rate is simply given by 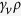 with 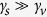. 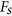 originates from a logistic law for birth and death and may vary with the strength of the chemotatic current (so 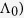 as shown in Fig. 1 on left (see also the discussion in the Supplementary Information).

We need now a mechanical law to evaluate the bacteria velocity 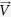 in Eq.(2). In a thin liquid layer of thickness *b* and viscosity *μ*, the horizontal fluid velocity is given by the Darcy law: 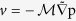, with a mobility coefficient 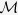 equal to 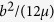. This assumption is valid for passive Newtonian suspension of spherical particles. However, kinetic microscopic models^9^,^10^,^36^ suggest that more appropriate choices may be made in case of rod-like particles, passive or active, giving explicit dependence of the viscosity with the particle density which differs from the Einstein relation. Concerning the dependence with controlled imposed shear-rates, Saintillan demonstrated^10^ that the viscosity increases for pushers (shear thickening behavior) and decreases for pullers (shear thinning behavior), at least in a suitable range of velocity values. This theory was experimentally confirmed at low shear-rates 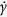, at least for *E. Coli* bacteria (a pusher)^12^ but a decrease of the viscosity after a critical 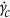 was also observed. In thin layers (flows in Hele-Shaw or thin films), the shear is mostly controlled by the small dimension *b*, its value being given by 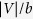 and the mobility coefficient is then transformed into a function of 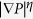. Such approximation gives a modified Darcy law^37^ for the averaged horizontal velocity which was successfully compared to experiments with non-newtonian fluids and adhesive elastomers^38^. So in the colony the hydrodynamic velocity is given by


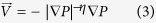



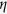
 being a trait of the bacterial activity. The coefficient 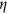 is negative for shear-thinning (corresponding to pushers), positive for a shear-thickening solution (corresponding to pullers) and the Newtonian case is represented by 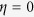. Also the flow outside satisfies Eq.(3) with 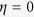. The pressure is normalized by the pressure unit: 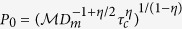, 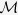 being the mobility coefficient (eventually modified by the rheology).

Patterns of bacteria result from the coupling of these 3 equations Eqs. (1, 2, 3), however Eq. (2) is difficult to analyze and is often simplified, the most common assumption being to neglect the velocity or to treat it as a perturbation as in^8^. Here, because of the radial expansion and the proliferation, we make the opposite choice and focus on an iso-density growing colony imposing 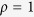 in Eq. (2) which gives us a sharp interface for the frontier between domains. Let us discuss this limit.

### An iso-density solution

An iso-density solution simplifies obviously the diffusion equation for morphogens as the mass-flux equation which becomes:


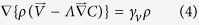


*ρ* being 1 in 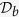, 0 in 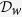. At the interface between domains 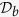 and 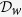 of normal 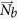, the continuity of the concentration and of the concentration gradient apply giving 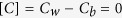 and 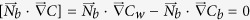. For the pressure field, the Laplace law of capillarity gives the gap across the interface. Calling *T* the surface tension, we have 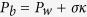 where 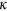 is the local curvature (positive for interfaces locally convex, negative otherwise). *σ* is the capillary parameter given by 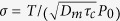, the unit of pressure 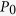 being defined above. The normal gradient of *P* involves the continuity of normal velocities: 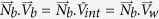, leading to


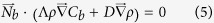


which proves that a gradient of bacterial concentration is required when there is a flux of chemo-attractant 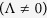. The immediate consequence of chemotaxis is then to make diffuse the interface, preventing sharp fronts in contradiction with our first hypothesis. However, as shown in numerical simulations^18^, it is possible to confine the diffusion on small scales compared to the size of 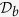 for a suitable adjustment of the proliferation rate (right-hand side of Eq.(2)). If this adjustment exits, it will allow to fix a thin boundary layer for the variation of the cell density *ρ*. Very often, diffuse interface models are used for numerically tracking front problems^39^,^40^,^41^, in our case and because of chemotaxis, the mechanical equilibrium of the interface imposes the existence of such boundary layer.

#### Density boundary layer at the frontier between domains

**Figure 2:**
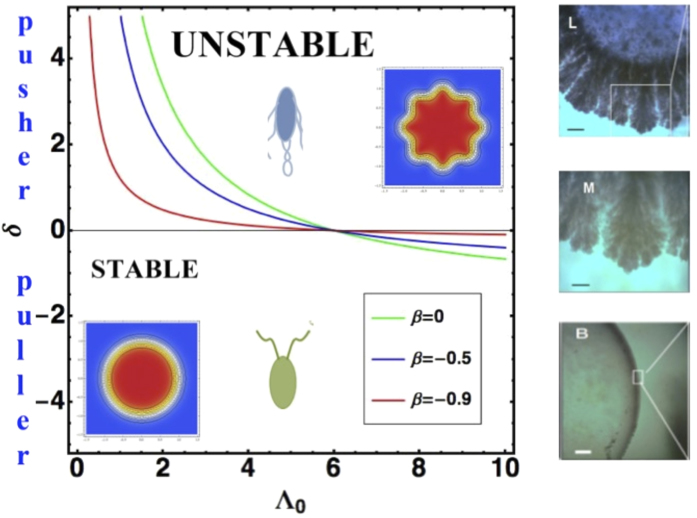
Limit of destabilization of the boundary-layer for active bacteria defined by the parameter *δ* = (*C*_*a*_/32)*L*^2^*b*^2^ <*n*_0_>/(*αR*_*b*_) (see Supplementary information) where *b* is the thickness of the colony, *L* the size of the bacterium including the bundle, <*n*_0_> the averaged bacteria number per unit volume, *R*_*b*_ the radius of the colony. *C*_*a*_ is the constant of anisotropy with absolute value around 0.57, positive for pushers, negative for pullers. Pushers destabilize the boundary layer for a 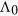 value smaller than 6 (the couple (*δ*, 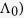 is then located above the curves corresponding to the viscosity contrast *β*). Pullers on contrary stabilize the boundary layer. On right, typical experimental bacterial patterns (M, N) of 14-old-day *E. coli* colony and their corona from^44^. For (L) magnification is 40, for M 100. On right below, the corona is tiny, stable and inhibited by glucose contrary to *L* and *M*.

Before going further, we examine the condition of existence of a tiny diffuse boundary layer of size *α* around a circular colony of radius 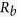 with 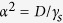 under the assumption that *α* is much smaller than the unit length and 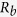. Defining 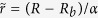, a local expansion in *α* transforms Eq.(2) locally in the moving front, giving the leading order cellular density 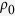 in the boundary layer as:


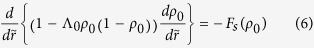


with 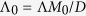, 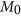 being the jump in concentration on both sides of the boundary layer: 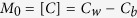. As shown in the Supplementary Information, the parameter 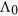 as 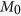 is fixed by 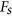, the surface proliferation rate, but it also exists a jump in the normal gradient 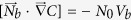 which physically means a source of chemo-attractants produced by the bacteria at the border. This process is called auto-chemotaxis, meaning that the bacteria produce their own morphogens and again, the interface exacerbates locally the morphogen production. These discontinuities remind us the case of impurity dendritic growth^42^ except that here the jump in morphogen concentration 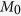 is not fixed by any thermodynamic consideration but by the biology of the bacteria. Even tiny, this boundary layer affects the concentration of morphogens as the pressure and velocity fields which will present discontinuities at the front. If this diffusive layer is stable (in practice for 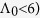, one can treat the pattern as a viscous fingering pattern. This explains why some bacteria colonies present strong similarities with viscous fingering experiments^43^. However, for active bacteria^8^, this layer may become unstable, destroying our picture of well separated domains. Indeed, active stresses created by active bacteria are dependent on density gradients so do not exist inside 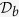 but they exist in the boundary layer. The viscous stresses that active bacteria exert on the flow contribute to a parameter *δ* which multiplies 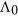, which then exceed the threshold of stability. This is the case for pushers but on the contrary pullers will stabilize the boundary layer (See Fig. 2). Our analysis predicts an anti-diffusive equation for the boundary layer and experimentally, strong nonlinear behavior of the corona (with spikes) is expected above the stability threshold *δ* in case of pushers as demonstrated by the experiments of^44^. Remember that *E. coli* is a pusher. Eq.(6) reinforces the similarity of our model and Cates *et al.* analysis^18^ where a similar structure is reached by introducing non-linearities in the diffusion coefficient *D*. Here no additional nonlinearities in the coefficient is required, the equation Eq.(6) only results from the proliferation rate. In the next section, we assume the front locally stable and solve the free-boundary problem of the colony growth, taking into account the change in the boundary conditions.

## Results

### Bacterial colony growth as a free boundary problem

Even if we adopt the simplified picture proposed by our model, the boundary conditions to apply to the interface leads to a complex free boundary problem which has been investigated in the past in the context of viscous fingering, dendritic growth and more generally pattern formation. The strategy consists in focusing on a simple geometry like the circular one and then to study its stability. In both domains 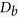 and 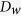, we first consider the morphogen concentration field varying instantaneously and neglect 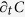 in Eq.(1) which is a common assumption. For large experimental set-ups with a typical radius 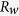, this approximation may be corrected by a cut-off for the diffusion field at long distances, but it remains valid in the interface vicinity. With the modified boundary conditions, we easily derive:


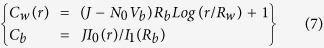



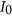
 being the modified Bessel function of zero order, regular at the origin. The concentration is fixed to 1 at 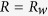. The parameter *J* is constrained by the jump in concentration 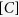 and is dependent of the time dependent value of the interface radius: 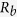. For small radius, if 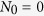, its value is given by 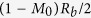 while for large radius, it becomes 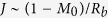 otherwise. Here the geometry imposes 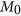 smaller than 1 since our basic solution assumes that a source of morphogens exist far away from the front. If the source of morphogens only originates from auto-chemotaxis, one needs to modify this base state without modification of the definition of *J*.

The velocity 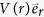 and the pressure *P* inside 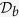 are then given by:


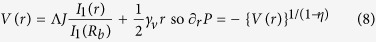


leading a growth velocity also depending on 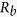. The dynamics of the interface results from 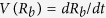 which gives an exponential growth for large 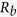, which automatically limits the validity of the circular solution in case of auto-chemotaxis and growth. Chemotaxis cannot induce alone the growth of the colony, even if it dominates the dynamics at short times. Taking into account earlier studies on viscous fingering^45^,^46^, a radial front has all the chances to be unstable and a more ramified pattern may be observed. In the following, we study the physical or biological parameters at the origin of the ramification.

### Instability Onset

We consider now the stability analysis of the circular solution in order to predict if this solution will be observed in practice or will be destabilize giving a undulated pattern. Such stability treatment^45^,^46^ also gives tendencies about the short time dynamics of the system, with an indication of the number of contour undulations observed at finite time. Non linear analysis based on the principle of the dissipation extremum has been performed for Darcy and Stokes flows^47^ and compared to the principle of extremum of the growth rate that we adopt here, for simplicity. In the case of multiple independent parameters, such treatment will allow to discriminate the physical parameters in favor of destabilization of the colony geometry. Indeed, the diversity of observed patterns requires to identify the parameters in favor of the stability versus the parameters in favor of destabilization. Then, we assume a wavy perturbation of the front, with a wavenumber *m*, inducing perturbations on both the nutrient concentration *C* and the pressure field *P* as:


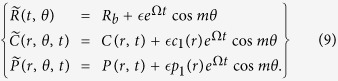


Solutions for the nutrient concentration become: 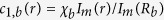 inside 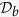 and 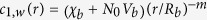 outside, 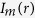 being the modified Bessel function of order *m* regular at the origin. The continuity of the morphogen flux along the normal of the front gives 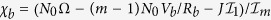, where 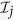 means the ratio between 2 successive Bessel functions evaluated at the front: 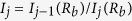. The solution for the pressure is more difficult to derive due to the nonlinearity of the p-Laplacian equation and the fact that Eqs. (42,43) (of the Supplementary Information) are also inhomogeneous. Approximate results can be found leading to:


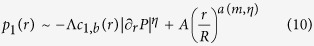



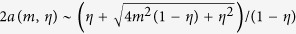
. The constant *A* is fixed by the Laplace law which gives the pressure jump at the front due to the surface energy: 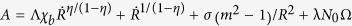, λ being the kinetic effect (see Eq. (26) of the Supplementary Information). Finally the dispersion relation is obtained by applying the modified Darcy Law: 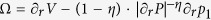. For simplicity here, we only give the results for 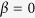, where both domains have the same viscosity, which is typical of the growth in a Petri-dish or in a thin film, and we also assume that the bacteria are passive 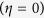. The more general case can be found in the Supplementary Information, Eq. (49). This case which simplifies the analysis does not lead to any instability in viscous fingering. Defining 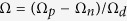, separating a unstable contribution 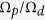 from the stable one 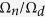 with 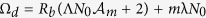, we derive:


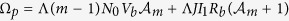


with 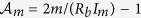 and


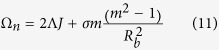


The negative (resp. positive) sign of Ω indicates a circular front which is stable (resp. unstable). If it is positive a complex pattern emerges. For small 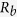 the spectrum is dominated by the stabilizing effect of surface tension becoming eventually unstable for large 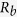 values (when the colony grows). Indeed, the asymptotic limit is easily found and given by: 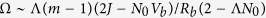. For 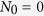, large 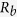 values lead to unstable patterns with or without growth but positive 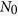 value and growth can stabilize the pattern. Increasing the viscosity contrast by changing the parameter *β* from 0 to −1 makes the pattern more stable, a physical result in opposition with viscous fingering. So colonies growing on an agar substrate^48^ are more stable, less ramified than colony growing in a liquid bath where 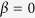. The same physical answer will concern an epithelium moving on a substrate. For shear-thinning solutions (the viscosity of the bacterial domain decreases with the velocity, *η* being negative), we again get a more unstable situation since 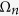 can becomes negative (see Eq (47) of the Supplementary Information).

**Figure 3:**
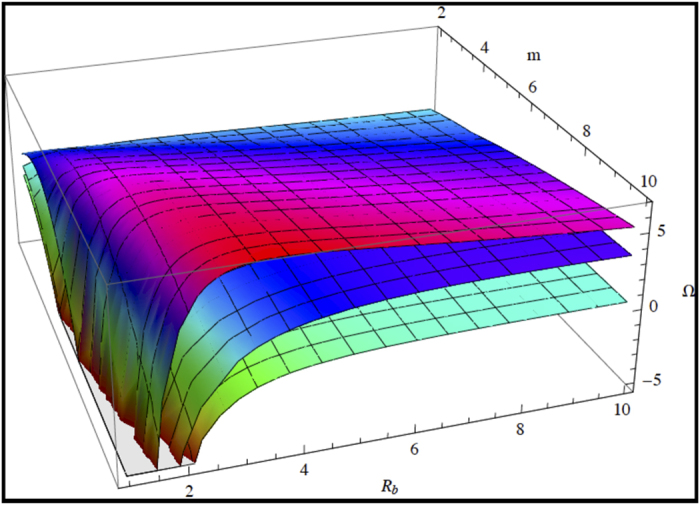
Growth rate of instability Ω as a function of *m* and *R*_*b*_. Comparison is done with 3 sets of data with growth corresponding to 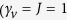, 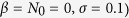. For the lowest sheet, 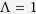, for the intermediate and upper sheets, 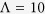, for the upper sheet, shear thinning is included and 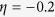. The first set of data indicates an instability only for large 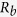 so appears when the colony grows, contrary to the 2 other cases where unstable modes occur for smaller values of 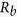.

**Figure 4:**
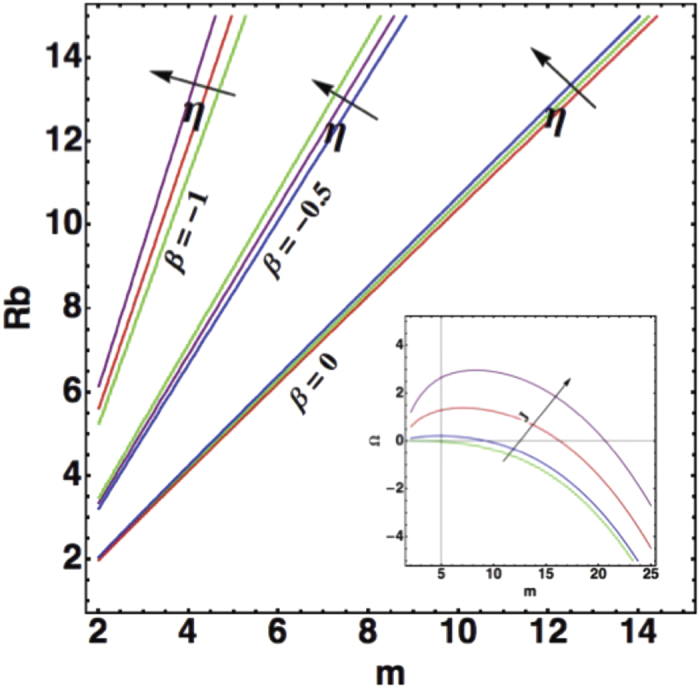
Mode Selection as a function of the radius in absence of growth. Selected modes are solution of 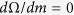, at fixed parameters: 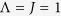, 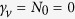. The viscosity contrast varies between 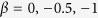 (0 indicates bacteria colony growing in a film or in a bath, 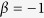 a circular domain expanding on a substrate). Notice the weak variation with *η*, the rheology coefficient. In the insert an example of the growth rate Ω as a function of *m* for 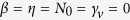 corresponding to Eq. (11).

**Figure 5:**
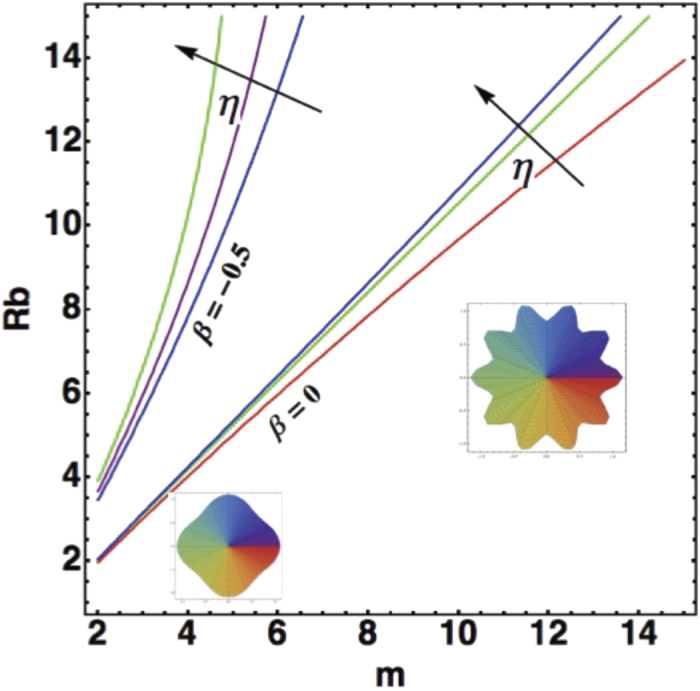
Mode Selection as a function of the radius with growth. Selected modes are given by 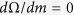, at fixed parameters: 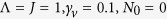. The viscosity contrast varies between 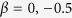. There is no instability modes for 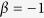.

Due to the multiplicity of parameters, we present on graphs the results of the dispersion relation when the parameters vary in Figs 3, 4, 5. In Fig. 3, we study the competition between growth and chemotaxis. Growth is a stabilizing process contrary to chemotaxis. We compare also the case with and without shear-thinning. Obviously, shear-thinning is exacerbated with growth at large 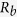 since the expansion is faster. In Fig. 4, we change the viscosity contrast between 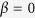 to 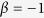 for 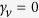, 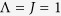 and we study the undulation number of the contour as a function of the colony size 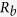. The undulation number increases when the viscosity contrast decreases, again a manifestation of the instability which occurs when the viscosity contrast vanishes and in opposition with traditional viscous fingering. Shear-thinning versus shear-thickening is not very sensitive because the velocity remains a constant in this case where the volume proliferation is not involved 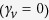. On the same graph, varying *J* from 0.1 to 10 and fixing the radius 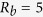 the inset shows that if *J* is too weak, there is no instability and the colony remains circular, when *J* is increased, the stability is lost, a maximum of Ω appears around a *m* value indicating the selected mode. If we now introduce 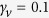 (see Fig. 5) with the same values of 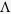 and *J*, the circular pattern remains stable for 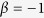 (maximum of viscosity contrast); for other values of the viscosity contrast, the instability increases with the rheology but only slightly, since the range of *η* values is limited between 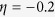 and 0.2.

To conclude, bacterial colony expansion has all the chance to exhibit complicated patterns under chemotaxis as soon as the size increases, even if the bacterial diffusion remains localized. Bacterial proliferation, which increases the velocity, stabilizes the core of the colony up to a certain point. However, as shown in Figs 4 and 5, there is a possibility to observe circular patterns, for a large viscosity contrast but also with shear-thickening.

## Discussion and Conclusion

Here we focus on growth expansion of dilute bacterial colony in the hydrodynamic regime. We pay special attention to chemotaxis coupled to proliferation and show that bacterial density gradient and hydrodynamics combine at the border of the colony. We choose as length unit a diffusive length constructed with the morphogen diffusion coefficient which is of order 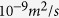 for oxygen for example^49^ and a capture time of order half an hour^50^ giving a typical length scale as the millimeter while velocities are of order *μ*m/s. It is a good estimation for the velocity expansion which is approximatively 1 cm/day as mentioned in^2^. Chemotaxis induces a boundary layer at the colony border whose relative thickness is given by the square-root of the relative bacterial diffusion coefficient *D* divided by the local proliferation rate. This coefficient *D* introduced in Eq.(2) can be derived knowing the swimming speed of bacteria about 20 *μ*m/s and the average time between tumbling events of order the second, giving 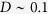
15. If the surface proliferation rate is of order 10, 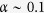, and the boundary layer will be of order 100 *μ* which is the typical thickness for destabilization of a thin film in case of active bacteria^7^. In the Supplementary Information, we discuss in detail the occurrence of such instability if the density of active bacteria is sufficient. Such instability superposes to the bulk fingering instability studied in the last part of this article and represented in Fig. 1 bottom right and we show that a weak contrast of viscosity increases the strength of the instability. The capillary parameter fixed to 0.1 in the numerics is quite irrelevant in experiments done in a Petri dish or thin film. Nevertheless, capillarity increases significantly for a drop put on an agar substrate, confirming the relative stability of this set-up^48^ against branching.

The simple continuous model of growing colonies proposed here aims to understand the branching instabilities observed in pioneering studies on bacterial colonies^1^,^2^. Branching patterns, not shown here, can be found in the literature and many models can give branching instabilities in radial geometry. It is clear that quantitative experiments are extremely useful to discriminate between the different approaches. The simplicity of our model comes from the fact that we do not introduce useless nonlinearities except perhaps in the modified Darcy law for active bacteria, which has been experimentally shown^12^. We couple a minimal set of equations and nutrients are simply hidden in the proliferation rate or eventually in the chemoattractant concentration. Contrary to earlier works, we focus on the hydrodynamics, an approach which is commonly adopted in tumorogenesis^22^,^23^. However, under chemotaxis, we show that density gradient cannot be ignored because of boundary conditions, which must verify the mechanical balance for soft interfaces. These density gradients may be at the origin of strong dynamical instabilities, exacerbated in case of active bacteria. However observations of experiments suggests that it may be possible to observe well separated domains^1^ suggesting perhaps the existence of a specific behavior of the bacteria proliferation at the front. Indeed we know that bacteria may have particular behavior at solid walls and interfaces, making necessary a modification of the boundary conditions^51^,^52^. These subtle modifications are not limited to active bacteria, although in this case the rotational dynamics of the swimming bacteria depends on the nature at solid walls^51^ or soft interfaces^52^. No such effect is taken into account here. Also the increase of bacterial density at solid walls in confinement has been demonstrated in^53^. This effect, if it exits for some interfaces may be introduced in the proliferation rates 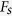 without difficulties. Among the physical properties in favor of compact patterns, we have identified first the proliferation 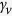, a strong viscosity contrast, the limiting case being colonies growing on agar and shear-thickening. Among the destabilizing properties we have chemotaxis, shear-thinning, and spatial expansion. Indeed it seems that branching always occurs but the core of the colony can be either compact or ramified.

## Additional Information

**How to cite this article**: Ben Amar, M. Collective chemotaxis and segregation of active bacterial colonies. *Sci. Rep.* **6**, 21269; doi: 10.1038/srep21269 (2016).

## Supplementary Material

Supplementary Information
