# Supplementary material for: Collective chemotaxis and segregation of active bacterial colonies
Source: Sci Rep. 2016 Feb 18;6:21269. doi: 10.1038/srep21269 (PMC4758065; doi:10.1038/srep21269)
Supplement: Supplementary Information [file srep21269-s1.pdf]

SUPPLEMENTARY INFORMATION: Collective chemotaxis and segregation of active bacterial colonies

M. Ben Amar
Laboratoire de Physique Statistique, Ecole Normale Supérieure, UPMC Univ Paris 06, Université Paris Diderot, CNRS, 24 rue Lhomond, 75005 Paris, France and Institut Universitaire de Cancérologie, Faculté de médecine, Université Pierre et Marie Curie-Paris 6, 91 Bd de l'Hôpital, 75013 Paris, France
(Dated: December 22, 2015)

1. INTRODUCTION

In the main manuscript, we describe two instabilities driven by chemotaxis: one arising at the border, the other in the interior. We show that the mass-flux equation (in its simplest version) produces a diffuse front when chemotaxis occurs, which may be abrupt otherwise. In addition, the flow induced in the advancing domain may present fingering instabilities, in the same way as viscous fingering in the same geometry. Both instabilities occur on different scales, the first one at the leading front which may remain tiny for a suitable adaptation of the proliferation rate compared to the driving instability factor, the other takes place everywhere in the colony space. If there is no adaptation of the proliferation at the border, the front becomes diffuse and a time dependent numerical treatment coupling velocity, morphogen and bacteria density changes is necessary [1, 2]. If this adaptation of the proliferation occurs, a front exits and we can consider the growth of a domain as a classical free-boundary growth problem, that we consider in the last section. In the immediate neighborhood of the interface, we will apply the asymptotic and local analysis made in classical phase-field model treatments [3], in the colony domain,we will apply the standard analysis of fingering.

2. DENSITY BOUNDARY LAYER AND MORPHOGEN CONCENTRATION

An iso-density pattern is an interesting starting point for analytical treatments and it gives immediately obvious similarities with Hele-Shaw flow dynamics. However, in case of chemotaxis, under this assumption, the correct boundary conditions cannot be satisfied, one of them (Eq.(5)) remaining mechanically unbalanced. Looking for an expanding growing domain under chemotaxis, an abrupt moving front is not rigorously valid although pattern observations [1, 2] may justify such hypothesis. It is why in this section, we introduce a boundary layer controlled by a sharp but continuous bacteria density front, between the colony domain $\mathcal{D}_b$ (see Fig.(1)) and the water domain $\mathcal{D}_w$.

A classical choice for the density inside the layer is given by $\rho = (1 - \tanh \tilde{r})/2$, where $\tilde{r} = (R - R_b)/\alpha$. $R - R_b$ is the distance from the interface and α, the ratio between the thickness of the boundary layer and the length unit. Let us remember that $\alpha = \sqrt{D/\gamma_s}$, with D the ratio between the bacteria and morphogen diffusion coefficient. γ_s gives the magnitude of the surface proliferation rate (see Eq.(2) of the *main text*), the bulk proliferation rate scaled by γ_v (Eq.(2)) being an order of magnitude smaller. The rescaled distance $\tilde{r}$ is positive (inside $\mathcal{D}_w$) defined from the middle of the boundary layer and negative inside $\mathcal{D}_b$. Clearly this sharp variation of the bacteria density will induce also a sharp variation of the morphogen concentration C, of the pressure P as well as the velocity fields $\vec{V}$. Inside the boundary layer, we will have to modify Eq.(1) and Eq.(2) of the *main text*, whose boundary conditions lead to the above inconsistency. The asymptotic analysis, presented here, is independent of the shape of the front since we can always define a local orthogonal coordinate system (r, s) where the unit vectors $(\vec{e}_r, \vec{e}_s)$ are respectively along the normal and the tangent. Asymptotically when $\tilde{r} \to \pm\infty$, the modifications of the concentration and pressure equations must correspond to Eq.(1) and Eq.(2), the inner solutions must recover the outer solutions for $R \to R_b$. In the following, we use capital letters for the far concentration field C (outer field) and small letter c (inner field) for its equivalent inside the boundary layer (the same for P and p). The concentration C verifies the following diffusion-consumption equation:

$$\frac{\partial C}{\partial t} + (M_0 - N_0)\frac{\partial \rho}{\partial t} = \nabla^2 (C + M_0 \rho) - \rho C \tag{1}$$

which asymptotically gives Eq.(1) when $\tilde{r} \to \pm\infty$. Let us consider now the inner expansion in the local coordinate system ($\vec{e}_r$, $\vec{e}_s$) shown in Fig.(1). In this system, along the interface, using the normal reduced $\tilde{r}$ coordinate and the arclength s, the Laplace operator and the time derivative applied to c give the following relations:

$$\begin{cases} \Delta C = \frac{1}{\alpha^2}\frac{\partial^2 c}{\partial \tilde{r}^2} + \kappa \frac{1}{\alpha}\frac{\partial c}{\partial \tilde{r}} + |\nabla s|^2 \frac{\partial^2 c}{\partial s^2} + \kappa \frac{\partial c}{\partial s} \\ \frac{\partial C}{\partial t} = \frac{\partial c}{\partial t} + \frac{1}{\alpha}(\partial_t r)\frac{\partial c}{\partial \tilde{r}} + (\partial_t s)\frac{\partial c}{\partial s} \end{cases}$$

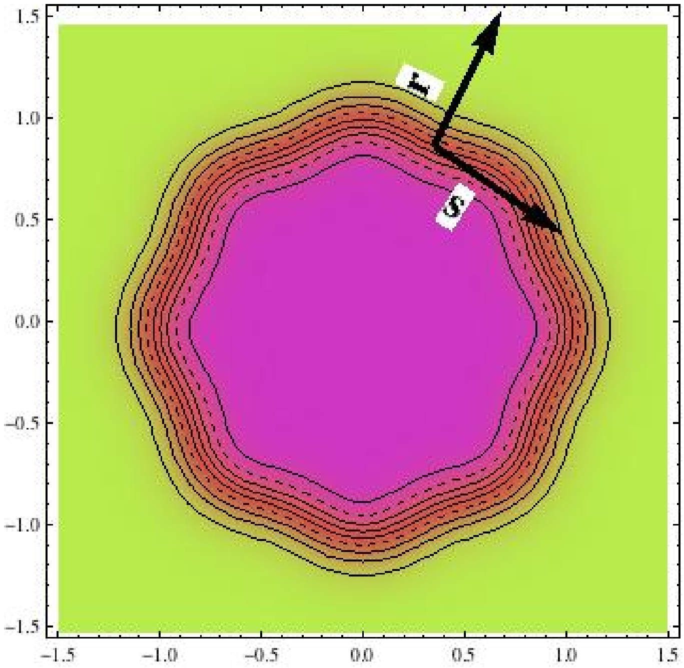

FIG. 1: Representation of the colony expansion with a gradient of bacterial density. In pink the bacterial domain $\mathcal{D}_b$, in green $\mathcal{D}_w$. The curvilinear coordinates $\tilde{\mathbf{r}}$ and $\mathbf{s}$ are represented.

where κ is the local curvature of the front (positive when it is convex) and $\partial_t r = -V_b$. In the boundary layer, Eq.(1) is then transformed into:

$$\frac{\partial^2 (c + M_0\rho)}{\partial \tilde{r}^2} + \alpha\kappa \frac{\partial (c + M_0\rho)}{\partial \tilde{r}} + \alpha(M_0 - N_0)V_b \frac{\partial \rho}{\partial \tilde{r}} + \alpha V_b \frac{\partial c}{\partial \tilde{r}} + O(\alpha^2) = 0 \tag{2}$$

Solving this equation for c order by order reads:

$$c = c_0 + \alpha c_1 + \alpha^2 c_2 + ... \text{ and } \rho = \rho_0 + \alpha\rho_1 + \alpha^2 \rho_2 + ... \tag{3}$$

and we derive to leading order:

$$c_0 = -M_0(\rho_0 - 1) + C_b(s) \tag{4}$$

where C_b is the far-field value of the concentration, eventually a function of the arclength s f. Notice that M_0 is related to the concentration jump at the interface since

$$c_0|_{+\infty} - c_0|_{-\infty} = C_w - C_b = M_0 \tag{5}$$

which is the Dirichlet condition. Considering the next order, we get:

$$\frac{\partial}{\partial \tilde{r}}(c_1 + M_0\rho_1) = N_0 V_b(\rho_0 - 1) \tag{6}$$

which gives for $\tilde{r} \to \pm\infty$

$$[C']_b^w = C'_w - C'_b = -N_0 V_b \tag{7}$$

Eq.(7), which is the Neumann relation, corresponds to a jump in the normal gradient, N_0 playing the role of the latent heat in dendritic growth. Contrary to dendritic growth of alloys [3] where the jumps at the level of the Neumann Eq.(7), and the Dirichlet, Eq.(5), conditions are dictated by thermodynamics, at this stage M_0 and N_0 are postponed. However, the similarity with dendritic growth indicates the physical meaning of N_0 being the auto-chemotaxis rate of production by unit time and volume.

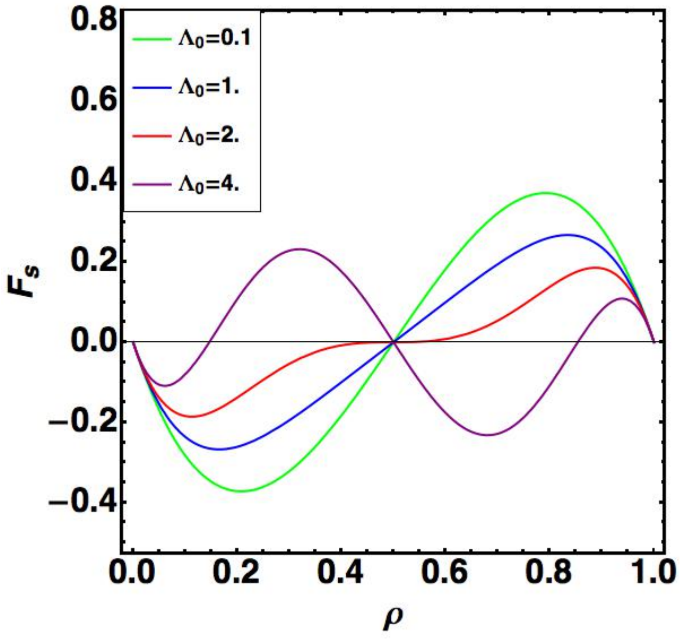

FIG. 2: Surface proliferation rate $F_s(\rho)$ as a function of the bacterial density ρ, according to Eq.(15). $F_s = -4\rho(1-\rho)(1-2\rho)(1-2\Lambda_0\rho(1-\rho))$ and the density profile, represented in the inset is $\rho = (1-\tanh(\tilde{r}))/2$. Notice for $\Lambda_0 = 4$, the strange behavior of the density function confirming that $\Lambda_0 = 4$ is the limit of the stability.

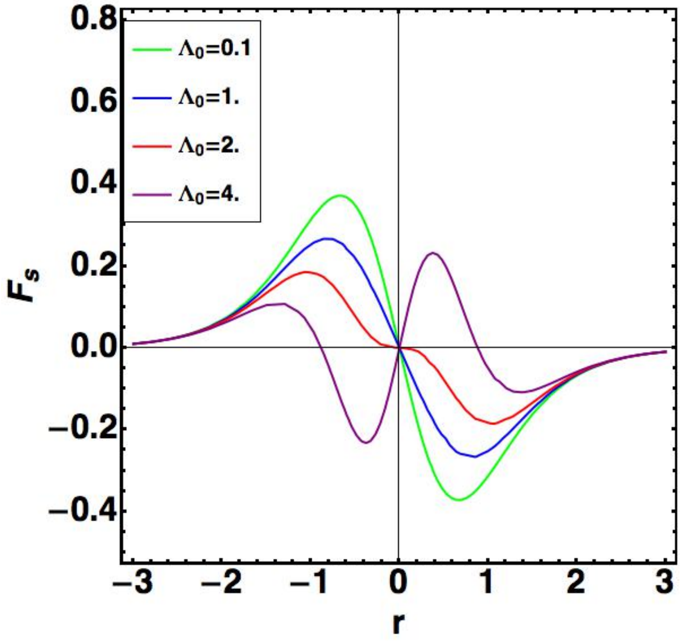

FIG. 3: Surface proliferation rate $F_s(\rho)$ as a function of the bacterial density ρ, according to Eq.(15). $F_s = -4\rho(1-\rho)(1-2\rho)/(1-\Lambda_0\rho(1-\rho))$ and the density profile, represented in the inset is found numerically. The same code of colors applies to the inset. A singularity occurs here for $\Lambda_0 = 4$.

3. DENSITY BOUNDARY LAYER AND THE PRESSURE EQUATION

In the same way, we consider a continuous version of the velocity equation:

$$\vec{\nabla}.(\vec{v} - \rho\Lambda\nabla c) \text{ where } \vec{\nabla}.\vec{v} = \frac{1}{\alpha}\frac{\partial v_r}{\partial \tilde{r}} + \kappa v_r + \frac{\partial v_s}{\partial s} + v_s\kappa \tag{8}$$

For passive bacteria, we represent the velocity field by the gradient of the pressure deduced from Darcy law (here for simplicity, we restrict on Newtonian hydrodynamics) and we add to the pressure a density dependent contribution, different in the bacteria ($\mathcal{D}_b$) and the water ($\mathcal{D}_w$) baths:

$$v_r = -\frac{1}{\alpha}\mathcal{M}\frac{\partial(p + \alpha\rho T(s)}{\partial \tilde{r}} \text{ and } v_s = -\mathcal{M}\frac{\partial(p + \alpha\rho T(s))}{\partial s} \tag{9}$$

which gives a more simple scalar equation for the pressure field coupled to the concentration field such that :

$$\nabla\{\mathcal{M}\nabla(p + \alpha\rho T(s)) + \rho\Lambda\nabla c\} = 0 \tag{10}$$

where the mobility coefficient $\mathcal{M} = \frac{1+\beta\rho}{1+\beta}$. According to the Einstein relation, passive bacteria solutions are more viscous than water and correspond to $\beta < 0$. Positive values of β indicate that the bacteria are active. Expanding p as c in power of α in the local coordinate frame, we get to zero order:

$$\mathcal{M}\frac{\partial p_0}{\partial \tilde{r}} + \Lambda\rho_0\frac{\partial c_0}{\partial \tilde{r}} = \mathcal{M}\frac{\partial p_0}{\partial \tilde{r}} - \Lambda M_0\rho_0\frac{\partial \rho_0}{\partial \tilde{r}} = 0 \text{ so } p_0 = -\Lambda M_0\int_\rho^1 \frac{\rho}{\mathcal{M}(\rho)}d\rho \tag{11}$$

This relation shows that it exists a pressure jump due to chemotaxis. For our mobility choice, the jump in pressure is : $[P]_b^w = P_w - P_b = -\Lambda M_0(1+\beta)(\beta - Log(1+\beta))/\beta^2$. The next order reads:

$$\{\mathcal{M}\frac{\partial}{\partial \tilde{r}}(p + \rho_0 T(s))\}_1 = -V_b + \Lambda M_0\frac{\partial(\rho_0\rho_1)}{\partial \tilde{r}} - N_0\Lambda V_b\rho_0(\rho_0 - 1) \tag{12}$$

At infinity, for $\tilde{r} \to \pm\infty, \partial\rho_1/\partial\tilde{r} = 0$ as $\rho_0(\rho_0 - 1)$. Then, the continuity of the normal velocity: $v_r = V_b$ is checked in both domains as physically required. To conclude, our model for the boundary layer is consistent with the continuity of the normal velocities and with $v_r = V_b$, but the pressure field is discontinuous at zero order.

4. THE MASS FLUX EQUATION

Taking into account Eq.(8), the mass flux equation is reduced to

$$\nabla\big((\rho - 1)\mathcal{M}\nabla p - \alpha\mathcal{M}\nabla(\rho T(s)) + D\nabla\rho\big) = -D(F_s(\rho) + \alpha\gamma\rho) + \alpha^2\frac{\partial\rho}{\partial t} \tag{13}$$

where $\gamma = \gamma_v/\sqrt{\gamma_s D}$. To leading order, it reads:

$$\frac{d}{d\tilde{r}}(1 - \Lambda_0\rho_0(1 - \rho_0))\frac{d\rho_0}{d\tilde{r}} + F_s(\rho_0) = 0 \tag{14}$$

where $\Lambda_0 = (\Lambda M_0)/D$. We require that $\rho_0(r)$ behaves like a kink with or without chemotaxis. Obviously it will depend on the function $F_s(\rho_0)$ which must cancel for $\rho_0 = 0$ and $\rho_0 = 1$. This equation can be solved exactly for arbitrary F_s function by defining $d\rho/dr$ as a function of ρ transforming the nonlinear second order O.D.E, Eq.(14), into 2 linear O.D.E of first order and we get:

$$\big(\frac{d\rho_0}{d\tilde{r}}\big)^2 = \frac{-2}{[1 - \Lambda_0\rho_0(1 - \rho_0)]^2}\int_0^{\rho_0} F_s(w)(1 - \Lambda_0 w(1 - w))dw \tag{15}$$

Eq.(15) shows that the bacteria repartition in the boundary layer is entirely determined by the surface proliferation rate $F_s(\rho)$. This function scaled by $\gamma_s \sim 1/\alpha^2$ must vary sharply at the front to provide a controlled size of the front.To be a kink solution requires that, in Eq.(15), the integral vanishes for $\rho_0 = 1$. Imposing that the kink exists with and without chemotaxis gives $\int_0^1 F_s(w)dw = 0$ in addition to the cancellation of $\int_0^1 F_s(\rho)(1 - \Lambda_0\rho(1 - \rho))d\rho$. So 3 options are possible:

1) A kink solution pre-exits without chemotaxis and $F_s(\rho)$ vanishes for $\rho = 0$ or 1. The bacteria do not change their proliferative strategy but the density ρ is modified. Assuming F_s is fixed one can suspect a singularity for $\Lambda_0 \geq 4$.

2) Due to chemotaxis, F_s is modified. It means that chemotaxis affects the proliferation rate.

3) The chemotaxis destroys the front making it more and more diffuse as time goes on.

A standard function for F_s giving a kink solution [7] is $F_s = G(W)(1-2\rho)$ with $W = \rho(1-\rho)$ and $G(0) = 0$. It will give a kink solution whatever the value of Λ_0, provided that the integral in the right-hand-side of Eq.(15) remains always negative. Considering hypothesis 1, and making the traditional and simple choice as $F_s = -4W(1-2\rho)$, we get in absence of chemotaxis $\rho = (1-\tanh(\tilde{r}))/2$ which corresponds to the solution in usual phase-field models, derived from the Landau approach for phase-transition. With chemotaxis, it becomes difficult to perform a direct integration of Eq.(15) and a numerical analysis is required.

For hypothesis 2, the transformation of the proliferative rate into $F_s = -4W(1-2\rho)(1-2\Lambda_0 W)$ gives the kink solution $(1-\tanh(\tilde{r}))/2$ (see Fig.(2), where F_s is represented) . The parameter Λ_0 is fixed by the proliferation rate and is not an unknown. It is not the only choice. For example, we can choose $F_s = -4W(1-2\rho)/(1-\Lambda_0 W)$ and in this case we get : $-\Lambda_0\rho + Log\rho/(1-\rho) = -2\tilde{r}$. All values of Λ_0 are possible, but these values so M_0 are fixed by the proliferation rate at the front separation (see F_s and the associated profile functions in Fig.(2)) .

Let us consider the next order in α. We first introduce the linear second-order differential operator:

$$\mathcal{L}(\rho_1) = \frac{d^2}{d\tilde{r}^2}\{(1-\Lambda_0\rho_0(1-\rho_0))\rho_1\} + F_s'\rho_1 \tag{16}$$

Using Eq.(12) we derive, after simplification,

$$\mathcal{L}(\rho_1) = -\kappa\{1-\Lambda_0\rho_0(1-\rho_0)\}\frac{d\rho_0}{d\tilde{r}} + \frac{N_0\Lambda_0}{M_0}V_b\frac{d}{d\tilde{r}}\{\rho_0(1-\rho_0)^2\} - \gamma\rho_0 + \frac{T(s)}{D}\frac{d}{d\tilde{r}}\big(\mathcal{M}\rho_0\frac{d\rho_0}{d\tilde{r}}\big) \tag{17}$$

$\mathcal{L}(\rho_1) = 0$ accepts as a solution $\rho_1 = d\rho_0/d\tilde{r}$, which can be checked by simple differentiation of Eq.(14) and whose adjoint is simply $\Phi_0 = \big(1-\Lambda_0\rho_0(1-\rho_0)\big)d\rho_0/d\tilde{r}$. A necessary condition for the convergence of the expansion in α is given by the adjoint theorem and reads:

$$\frac{N_0\Lambda_0}{M_0}V_b\int_{-\infty}^{\infty}\frac{d}{dy}\{\rho_0(1-\rho_0)^2\}\Phi_0(y)dy + \frac{T(s)}{D}\int_{-\infty}^{\infty}\frac{d}{dy}\big(\mathcal{M}\rho_0\frac{d\rho_0}{dy}\big)\Phi_0(y)dy$$
$$+\gamma\int_0^1\big(1-\Lambda_0\rho_0(1-\rho_0)\big)\rho_0 d\rho_0 - \kappa\int_{-\infty}^{\infty}\Phi_0(y)^2dy = 0 \tag{18}$$

Integrating the first integral by part, noticing that:

$$\Phi_0' = -F_s(\rho_0) \tag{19}$$

and introducing the following integrals:

$$\mathcal{J}_0 = \int_0^1 \mathcal{M}\rho F_s(\rho)d\rho\,,\ \ \mathcal{J}_1 = \int_{-\infty}^{\infty}\rho_0(1-\rho_0)^2F_s(\rho)dy \ \text{ and } \ \mathcal{J}_2 = \int_0^1\{1-\Lambda_0\rho(1-\rho)\}\rho d\rho \tag{20}$$

we get

$$\frac{T(s)}{D}\mathcal{J}_0 - V_b\frac{N_0\Lambda_0}{M_0}\mathcal{J}_1 - \gamma\mathcal{J}_2 = -\kappa\int_{-\infty}^{\infty}\Phi_0(r)^2d\tilde{r} = -\kappa\tilde{\sigma} \tag{21}$$

$\tilde{\sigma}$ being the capillary parameter as defined usually in phase-field models and $2\mathcal{J}_2 = 1-\Lambda_0/6$. This solvability condition gives a relation between the jump in concentration gradient N_0, the jump in concentration M_0, the jump in pressure $T(s)$ due to the proliferation rate represented by γ when the front keeps its kink shape.

When the surface proliferation rate defined in Eq.(2) of the main manuscript allows such kink formation it is possible to treat Eq.(2) as a free-boundary problem provided that suitable boundary conditions are applied for the continuous field. In absence of chemotaxis $\Lambda_0 = 0$, the coupling between morphogens and bacteria disappear but a diffuse front may exist. In this case we have:

$$\frac{T(s)}{D}\mathcal{J}_0 = \gamma/2 - \kappa\tilde{\sigma} \tag{22}$$

and the pressure jump at the interface, equivalent to Laplace law, becomes:

$$[P]_b^w = P_w - P_b = \Delta P_0 + \alpha T(s) = \Delta P_0 + \alpha D(\gamma/2 - \kappa\tilde{\sigma})/\mathcal{J}_0 = \Delta P_0 + \frac{1}{2}\frac{D}{\mathcal{J}_0}\frac{\gamma_v}{\gamma_s} - \sigma\kappa \tag{23}$$

The sign of κ has been chosen positive here for convex interface in agreement with the Laplace law, the pressure inside being larger than outside according to this law, in absence of growth. However, there is also a constant jump of pressure due to the volumetric growth.

5. PARTICULAR CHOICES FOR THE PROLIFERATION RATE

According to hypothesis (1), we fix the proliferation rate F_s to its value in absence of chemotaxis. Then the border proliferation rate is given by $4\rho_0(1-\rho_0)(2\rho_0-1)$, and we can calculate only $\mathcal{J}_0$ exactly. It does not depend on ρ_0 (difficult to evaluate), contrary to $\mathcal{J}_1$ and σ. In absence of chemotaxis having $\rho_0 = (1-\tanh(\tilde{r}))/2$, all integrations can be done and we get:

$$\mathcal{J}_0 = 1/15; \quad \mathcal{J}_1 = -1/30 \;\text{ and }\; \sigma = 1/3 \tag{24}$$

A linear expansion for weak Λ_0 value can be achieved for $\mathcal{J}_1$ and σ. For hypothesis (2) with $F_s = 4w(2\rho_0-1)(1-2\Lambda_0 w)$, being $w = \rho_0(1-\rho_0)$ and $\rho_0 = (1-\tanh(\tilde{r}))/2$, all integrals can be achieved exactly giving:

$$\mathcal{J}_0 = 1/15 - 2\Lambda_0/105; \quad \mathcal{J}_1 = -1/30(1-2\Lambda_0/7) \;\text{ and }\; \sigma = 1/3 - 2\Lambda_0/15 + \Lambda_0^2/70 \tag{25}$$

Adding chemotaxis modifies the Laplace relation giving the so-called kinetic effects of dendritric growth and a modification of the surface tension:

$$[P]_b^w = P_w - P_b = \Delta\Pi_0 - \sigma\kappa - \lambda N_0 V_b \;\text{ with }\; \lambda = \Lambda_0 \frac{D}{M_0}|\mathcal{J}_1|)/\mathcal{J}_0 \tag{26}$$

$\Delta\Pi_0 = \Delta P_0 + \frac{1}{2}\frac{D}{\mathcal{J}_0}\frac{\gamma_v}{\gamma_s}$ is a constant related to the growth. As expected, the sign of the *kinetic effect* λ is negative since $\mathcal{J}_1$ is negative behaving as a stabilizing effect as the surface tension.

6. STABILITY OF THE BOUNDARY LAYER VERSUS TIME DEPENDENT PERTURBATIONS

We perform now a stability analysis of the boundary layer in the spirit of Kasyap and Koch [4, 5] treatment for passive and active bacteria. We focus first on the passive case, the analysis being rather technical in our case since, first, the layer is curved and, second, has no fixed boundaries. We consider a dynamical perturbation of amplitude ϵ, ϵ being smaller than 1 and we solve the dynamics up to order α^2. The relevant physical quantities are then:

$$\begin{aligned} &R(t,s) = R_b + \epsilon e^{\Omega t} e^{iks} \\ &C = c^{(st)}(\tilde{r}) + \epsilon\tilde{c}(\tilde{r}) e^{\Omega t} e^{iks} \\ &P = p^{(st)}(\tilde{r}) + \epsilon\tilde{p}(\tilde{r}) e^{\Omega t} e^{iks} \\ &\rho = \rho^{(st)}(\tilde{r}) + \epsilon\tilde{\rho}(\tilde{r}) e^{\Omega t} e^{iks} \end{aligned} \tag{27}$$

Here, a steady state variable is mentioned by the label exponent (st) contrary to a dynamical one represented by a capital letter (except ρ). Dynamical quantities will be expended in modes up to linear order in ϵ. These modes are due to the waviness of the center line of the boundary layer (see Fig.(1)). Contrary to usual stability analysis and to the work by Kasyap and Koch [4], we only know the steady state $\rho^{(st)}, P^{(st)}, C^{(st)}$ up to order α which adds a degree of difficulty to the following treatment. From Eq.(13) we derive:

$$\frac{\partial\Pi_r}{\partial\tilde{r}} + \alpha\kappa\Pi_r + \alpha\frac{\partial\Pi_s}{\partial s} + \alpha\kappa\Pi_s = \frac{D}{\alpha}(-F_s(\rho) + \alpha\gamma\rho) - V_b\frac{\partial\rho}{\partial\tilde{r}} + \alpha\frac{\partial\rho}{\partial t} \tag{28}$$

with

$$\Pi_r = (1-\rho)v_r - T(s)\mathcal{M}\frac{\partial\rho}{\partial\tilde{r}} + \frac{D}{\alpha}\frac{\partial\rho}{\partial\tilde{r}} \tag{29}$$

$$\Pi_s = (1-\rho)v_s - a\mathcal{M}\frac{\partial(T(s)\rho)}{\partial s} + D\frac{\partial\rho}{\partial s} \tag{30}$$

Making the expansion in ϵ and taking the average according to the definition $< f >= \int_{-\infty}^{\infty} f(y)dy$ simplifies Eq.(28) by eliminating the first 2 terms of the left-hand-side combined with the first 3 terms of the right-hand side and giving $\tilde{\rho} = d\rho^{(st)}/d\tilde{r}$, the analysis being similar to the determination of ρ_1 given in the previous section. So we get

$$< \frac{\partial \tilde{\Pi}_s}{\partial s} > +\kappa < \tilde{\Pi}_s >= \Omega < \tilde{\rho} > \tag{31}$$

Considering modes having a wavelength smaller than the radius of curvature of the boundary eliminates the second term of the left-hand side of Eq.(31). Let us evaluate $\tilde{\Pi}_s$ knowing that to leading order we have: $v_s = -\mathcal{M}\frac{\partial p}{\partial s} = -\Lambda_0 D\rho\frac{\partial \rho}{\partial s}$, we obtain:

$$\Pi_s = D(1 - \Lambda_0\rho(1-\rho))\frac{\partial \rho}{\partial s} \tag{32}$$

giving

$$\tilde{\Pi}_s = ikD(1 - \Lambda_0\rho^{(st)}(1-\rho^{(st)}))\tilde{\rho} - D\Lambda_0(1 - 2\rho^{(st)})\frac{\partial \rho^{(st)}}{\partial s}\tilde{\rho} \tag{33}$$

Using again the hypothesis of large k value compared to relative variation of $\rho^{(st)}$ we finally get:

$$< \frac{\partial \tilde{\Pi}_s}{\partial s} >= -k^2 D < (1 - \Lambda_0\rho^{(st)}(1-\rho^{(st)})\tilde{\rho} >= k^2 D \int_0^1 (1 - \Lambda_0\rho(1-\rho))d\rho \tag{34}$$

which gives

$$\Omega = -k^2 D\mathcal{J}_2 \tag{35}$$

The stability of the boundary layer requires $\mathcal{J}_2 = 1 - \Lambda_0/6 > 0$ which limits the value of Λ_0. For $\Lambda_0 < 6$, the boundary layer is stable versus perturbations of short wavelengths. However, it does not mean that the boundary layer is stable for arbitrary wavelength. Indeed it depends on the proliferation rate itself which can be dependent of the arclength and of the local curvature. In this case, an instability mode may exist at finite k.

7. STABILITY ANALYSIS OF THE BOUNDARY LAYER WITH ACTIVE BACTERIA

A continuous model of active bacteria flow has been proposed by Kasyap and Koch [4] who show that the random walk of these bacteria is perturbed by the chemotacting forcing imposed transversally. It results an anisotropic and active stress in the flow which differs according they are pushers like *E.Coli* or *B. Subtilis* or pullers. Assuming a constant chemotacting forcing, represented by a velocity U_0 which induces a variation of bacteria density in the film, they prove the existence of a bio-convection process, recovering the experimental results of Sokolov and Aranson [6]. For weak concentration of chemicals and for dilute bacterial solutions, it is possible to average the bacterial motion on time scale larger than the time separation between 2 tumbling events $T \sim 1s$ and on a length scale larger than $V_p T$ (V_p being the individual bacterium velocity) and the Stokes equation (in their case) turns out to be modified by an active stress, average of the force-dipole interaction.

The active stress per bacterium is $S = -C_a/16\mu V_p L^2 \zeta(\mathbf{e_r} \cdot \mathbf{e_r} - 1/2\mathbf{I})$, with $\zeta = \alpha\Lambda V_p \partial_r c$ a coupling parameter with the chemotactic gradient, C_a is a geometrical factor, positive for pullers, negative otherwise, estimated to be $|C_a| \sim 0.57$, L means the effective size of the bacteria (taking into account the bundle). Individual velocity of bacteria V_p is larger than the flow velocity given by v_r and $V_p\zeta \sim 6v_r$. Defining a new parameter δ as the ratio between the average active stress and the pressure at zero order in the boundary layer leads to $\delta = (3C_a/8)\mu v_r L^2 \alpha < n_0 > /(12\mu v_r \alpha R_b/b^2)$, with R_b the radius of curvature of the pattern, b the thickness of the film or the Hele-Shaw cell, $< n_0 >$ the average number of bacteria per unit volume. So we get

$$\delta \sim (3C_a/8)L^2 b^2 < n_0 > /(12\alpha R_b)$$

.

One can estimate this parameter taking $L \sim 12\mu$m, $b \sim 10^{-3}$m, $R_b \sim 10^{-2}$m, $\alpha \sim 10^{-1}$ which gives : $\delta \sim 0.510^{-14} < n_0 >$. In Sokolov *et al.* experiments $< n_0 >$ is of order 10^{16} with a film thickness of order the μ. So δ is a parameter of order 1 or even larger. Because of the anisotropic active stress, the Darcy law is transformed into :

$$v_r = -\mathcal{M}\{\frac{\partial p}{\partial \tilde{r}} - \frac{\delta}{2}\Lambda M_0 \frac{\partial}{\partial \tilde{r}}(\rho^2 \frac{\partial \rho}{\partial \tilde{r}})\} \tag{36}$$

which does not affect our treatment if we define an effective pressure $\mathcal{Q} = p(r) - \frac{\delta}{2}\Lambda M_0 \rho^2 \frac{d\rho}{d\tilde{r}}$, playing exactly the same role as p. Then we get for the tangential velocity:

$$v_s = -\mathcal{M}\{\frac{\partial \mathcal{Q}}{\partial s} + \delta \frac{\partial}{\partial s}(\rho^2 \frac{d\rho}{d\tilde{r}})\} \tag{37}$$

with a change of sign in front of δ because of the anisotropy of the active stress. It modifies Eq.(32) into:

$$\Pi_s = D\{(1 - \Lambda_0 \rho(1-\rho))\frac{\partial \rho}{\partial s} - \delta \Lambda_0 \frac{1+\beta\rho}{1+\beta}(1-\rho)\frac{\partial}{\partial s}(\rho^2 \frac{d\rho}{d\tilde{r}}) \tag{38}$$

giving

$$\tilde{\Pi}_s = ikD[(1 - \Lambda_0(\rho^{(st)}(1-\rho^{(st)}))\tilde{\rho} - \Lambda_0 \delta \frac{1+\beta\rho^{(st)}}{1+\beta}(1-\rho^{(st)})\frac{\partial {\rho^{(st)}}^2 \tilde{\rho}}{d\tilde{r}}] \tag{39}$$

With our choice for the kink solution we get for :

$$< \frac{\partial \tilde{\Pi}_s}{\partial s} >= -k^2 D < (1 - \Lambda_0 \rho^{(st)}(1-\rho^{(st)}))\tilde{\rho} - \Lambda_0 \delta \frac{1+\beta\rho^{(st)}}{1+\beta}(1-\rho^{(st)})\frac{\partial {\rho^{(st)}}^2 \tilde{\rho}}{d\tilde{r}} >$$

which finally gives for the kink solution stability, the following growth rate for perturbations:

$$\Omega = -k^2 D\{1 - \frac{\Lambda_0}{6}(1 + \frac{\delta}{5}\frac{3+\beta}{1+\beta})\} \tag{40}$$

which increases the probability to get an instability of the boundary layer due to bio-convection in the case of pushers. The dispersion law, Eq.(40), have an effective diffusion coefficient which becomes negative for finite values of Λ_0 and δ. There is no prediction of the wavelength in this range of parameters. As shown in [7, 8], such a diffusive process may induce a spinodal decomposition in a full domain with domains of density 1 and others of density 0.

Up to now we focus our attention on the boundary layer which separates a quasi-homogeneous domain of bacteria and the bath containing the chemo-attractant. We show that chemotaxis is responsible of a diffusive layer and prohibits sharp front of separation. If the proliferation rate F_s at the level of the front satisfies some properties, the bacteria remain confined in a growing domain and the colony expands regularly. However this adjustment may fail in practice on long times. In addition, for active bacteria of type pushers and morphogen attractant, a wrinkling instability of the boundary layer itself may occur, mostly controlled by the bacteria density $< n_0 >$. In the next section, we will assume that it does not occur, the parameter Λ_0 being far from 6 and δ being small. This allows to transform the continuous set of equations into a free-boundary problem with the suitable boundary conditions for the morphogen concentration that we have found Eqs.(5,7).

8. STABILITY ANALYSIS OF THE FREE-BOUNDARY PROBLEM

The aim of this section is to study bulk instabilities, different from the one originated from the boundary layer (compare Fig.(4) with Fig.(1)). We consider the free-boundary problem in radial geometry, taking into account the correct boundary conditions derived previously. In radial geometry the zero-order is:

$$\left\{ \begin{array}{l} V = \Lambda J \frac{I_1(r)}{I_1(R_b)} + \frac{1}{2}\gamma_v r \text{ so } \partial_r P = -\{V(r)\}^{1/(1-\eta)} \text{ and } V_b = \Lambda J + \frac{1}{2}\gamma_v R_b \\ V_w = V_b R_b / r \text{ and } P_w = -(1+\beta) V_b R_b Log(r/R_b) \\ P_w - P_b = \Delta\Pi_0 - \sigma\kappa - \lambda N_0 V_{int} \text{ at } R = R_b \end{array} \right\} \tag{41}$$

The linear expansion requires the linear order of the morphogen concentration to calculate $v_1(r)$ as $p_1(r)$. Then, the continuity of the velocity and the discontinuity of the pressure at the perturbed interface: $R = R_b + \epsilon e^{\Omega t}\cos(m\theta)$ and normal velocity at the interface $V = V_b + \epsilon\Omega e^{\Omega t}\cos(m\theta)$ will allow to determine completely the flow field. But first let us evaluate the pressure field to linear order for a p-Laplacian according to Eq.(8) of the main manuscript. A first integral of the mass flux equation: $\nabla \cdot (V - \Lambda \nabla C) = 0$ leads to:

$$\begin{array}{l} \frac{\partial P}{\partial r} \cdot |\vec{\nabla} P|^{-\eta} + \Lambda \frac{\partial C_b}{\partial r} = -\frac{1}{r}\frac{\partial \Psi}{\partial \theta} \\ \frac{1}{r}\frac{\partial P}{\partial \theta} \cdot |\vec{\nabla} P|^{-\eta} + \Lambda \frac{1}{r}\frac{\partial C_b}{\partial \theta} = \frac{\partial \Psi}{\partial r} \end{array} \tag{42}$$

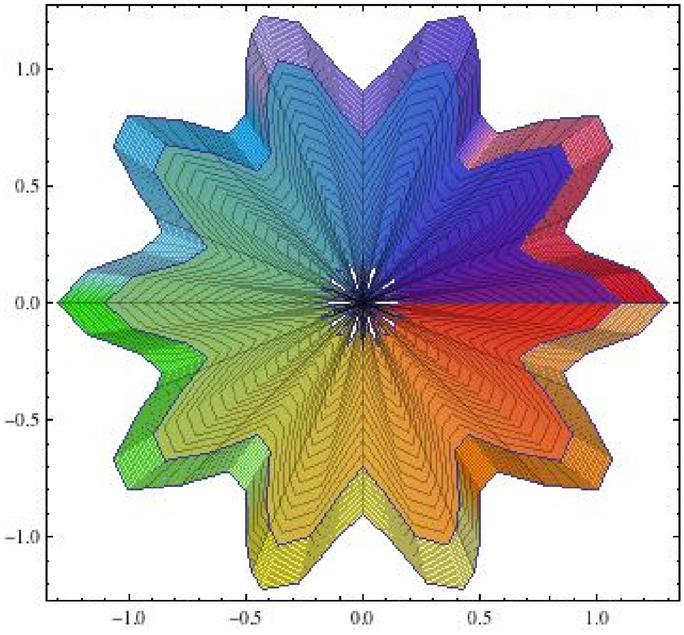

FIG. 4: Representation of the colony expansion with a bulk instability corresponding to Section (8). The boundary layer follows the instability of the bulk.

Ψ is an arbitrary function that we choose as $\Psi = \epsilon\psi(r)e^{\Omega t}\sin m\theta$ to expand Eq.(42) to linear order in ϵ. Then we get:

$$\left\{ \begin{array}{l} (1-\eta)\frac{dp_1}{dr} \cdot |\partial_r P|^{-\eta} + \Lambda\frac{dc_{1,b}}{dr} = -m\frac{\psi(r)}{r} \\ \\ -m\frac{p_1}{r} \cdot |\partial_r P|^{-\eta} - m\Lambda\frac{c_{1,b}}{r} = \frac{d\psi(r)}{dr} \end{array} \right\} \tag{43}$$

with $\partial_r P$ being given by the zero-order solution calculated previously:

$$\partial_r P = -V^{1/(1-\eta)} = -(\Lambda J\frac{I_1(r)}{I_1(R)} + \frac{1}{2}\gamma_v r)^{1/(1-\eta)} \tag{44}$$

Elimination of ψ is trivial by solving the first equation and replacing ψ into the second. Then we get the second order O.D.E for p_1:

$$(1-\eta)\frac{d}{dr}r|\partial_r P|^{-\eta}\frac{dp_1}{dr} - m^2|\partial_r P|^{-\eta}\frac{p_1}{r} = -\Lambda(\frac{d}{dr}r\frac{dc_{1,b}}{dr} - m^2\frac{c_{1,b}}{r}) \tag{45}$$

Although linear this equation cannot be solved exactly except for $\eta = 0$ which corresponds to the standard Darcy's equation. In this case we get

$$p_1(r) = -\Lambda c_{1,b}(r) + A(\frac{r}{R})^m \tag{46}$$

where $r^m Cos(m\theta)$ are the free modes which satisfy the Laplace equation. Another asymptotic solution can be found in the W.K.B limit for $m^2/(1-\eta)$ having a large value. Then we get to leading order:

$$p_1(r) \sim -\Lambda c_{1,b}(r)V_b^{\eta/(1-\eta)} + A(\frac{r}{R})^{a(m,\eta)} \tag{47}$$

with $2a(m,\eta) = (\eta + \sqrt{4m^2(1-\eta)+\eta^2})/(1-\eta)$. Defining Ω as

$$\Omega = \frac{\Omega_p - \Omega_n}{\Omega_d} \tag{48}$$

we can then separate the stabilizing effect giving a negative contribution to the growth rate Ω_n/Ω_d from the destabilizing effect with Ω_p/Ω_d. Of course it depends on the sign of Ω_d which is controlled by auto-chemotaxis.

$$\left\{\begin{array}{l} \Omega_p = \Lambda(m-1)N_0 V_b\big(\frac{a(m,\eta)+m}{\mathcal{I}_m(R_b)R_b} - 1\big) + \Lambda J \mathcal{I}_1(R_b)(\frac{a(m,\eta)+m)}{\mathcal{I}_m(R_b)} + \frac{\eta}{1-\eta}R_b) + 1/2\gamma_v \frac{R_b}{1-\eta} \\ \\ \Omega_n = a(m,\eta)V_b\big(1-(1+\beta)(1-1/m)V_b^{-\eta/(1-\eta)}\big) + \frac{\Lambda J}{1-\eta} + a(m,\eta)(m^2-1)V_b^{-\eta/(1-\eta)}\sigma/R_b^2 \\ \\ \Omega_d = \Lambda N_0 R_b(\frac{a(m,\eta)+m}{\mathcal{I}_m(R_b)R_b} - 1\big) + \frac{R_b}{1-\eta} + V_b^{-\eta/(1-\eta)}a(m,\eta)(R_b(1+\beta)/m + \lambda N_0) \end{array}\right\} \tag{49}$$

where as in the main manuscript, $\mathcal{I}_j$ means the ratio between 2 successive Bessel functions evaluated at the front: $\mathcal{I}_j = I_{j-1}(R_b)/I_j(R_b)$.

We consider now the case of an expanding drop or a moving epithelium sliding on a substrate. It means that our parameter $\beta \to -1$ to cancel the viscosity, outside the living matter domain. Then, the dispersion relation simplifies and reads:

$$\left\{\begin{array}{l} \Omega_p = \Lambda(m-1)N_0 V_b\big(\frac{a(m,\eta)+m}{\mathcal{I}_m(R_b)R_b} - 1\big) + \Lambda J \mathcal{I}_1(R_b)(\frac{a(m,\eta)+m)}{\mathcal{I}_m(R_b)} + \frac{\eta}{1-\eta}R_b) + 1/2\gamma_v \frac{R_b}{1-\eta} \\ \\ \Omega_n = a(m,\eta)V_b + \frac{\Lambda J}{1-\eta} + a(m,\eta)(m^2-1)\sigma V_b^{-\eta/(1-\eta)}/R_b^2 \\ \\ \Omega_d = \Lambda N_0 R_b(\frac{a(m,\eta)+m}{\mathcal{I}_m(R_b)R_b} - 1\big) + \frac{R_b}{1-\eta} \end{array}\right\} \tag{50}$$

---

[1] Ben-Jacob, E., Cohen I. & Levine H. Cooperative self-organization of microorganisms *Adv. Phys.* **49**, 395554 (2000)

[2] Ben-Jacob E. & Levine H. Self-engineering capabilities of bacteria. *J. R. Soc. Interface* **3**, 197214 (2006)

[3] Caginalp, G. & Xie, W . An analysis of phase-field alloys and transition layers,*Arch. Rational Mech. Anal* **142**, 293-329 (1998)

[4] Kasyap, T. V. & Koch, D.L. Chemotaxis driven Instability of a confined bacterial Suspension *Phys. Rev. Lett.* **108**, 03101-1-4 (2012)

[5] Kasyap, T. V. & Koch, D.L. Instability of an inhomogeneous bacterial suspension subjected to a chemo-attractant gradient *J. Fluid. mech* **741**, 619-657 (2014)

[6] Sokolov, A. & Aranson, I. R. Reduction of Viscosity in Suspension of Swimming Bacteria, *Phys. Rev. Lett.* **103**, 148101-1-4 (2009)

[7] Cates, M.E., Marenduzzoa, D., Pagonabarraga, I. & Tailleur, J. Arrested phase separation in reproducing bacteria creates a generic route to pattern formation *Proc. Nat. Acad. Sci* **107** (26) 1171511720 (2010)

[8] Chatelain, C., Balois, T., Ciarletta, P. & Ben Amar, M. Emergence of microstructural patterns in skin cancer: a phase separation analysis in a binary mixture *New Journ Physics* **13**, 115013-21 (2011)
